# Population differences in Chinook salmon (*Oncorhynchus tshawytscha*) DNA methylation: Genetic drift and environmental factors

**DOI:** 10.1002/ece3.7531

**Published:** 2021-05-01

**Authors:** Clare J. Venney, Ben J. G. Sutherland, Terry D. Beacham, Daniel D. Heath

**Affiliations:** ^1^ Great Lakes Institute for Environmental Research University of Windsor Windsor ON Canada; ^2^ Fisheries and Oceans Canada Pacific Biological Station Nanaimo BC Canada; ^3^ Department of Integrative Biology University of Windsor Windsor ON Canada

**Keywords:** adaptive potential, DNA methylation, epigenetics, genetic drift, population genetics, population variation

## Abstract

Local adaptation and phenotypic differences among populations have been reported in many species, though most studies focus on either neutral or adaptive genetic differentiation. With the discovery of DNA methylation, questions have arisen about its contribution to individual variation in and among natural populations. Previous studies have identified differences in methylation among populations of organisms, although most to date have been in plants and model animal species. Here we obtained eyed eggs from eight populations of Chinook salmon (*Oncorhynchus tshawytscha*) and assayed DNA methylation at 23 genes involved in development, immune function, stress response, and metabolism using a gene‐targeted PCR‐based assay for next‐generation sequencing. Evidence for population differences in methylation was found at eight out of 23 gene loci after controlling for developmental timing in each individual. However, we found no correlation between freshwater environmental parameters and methylation variation among populations at those eight genes. A weak correlation was identified between pairwise DNA methylation dissimilarity among populations and pairwise *F*_ST_ based on 15 microsatellite loci, indicating weak effects of genetic drift or geographic distance on methylation. The weak correlation was primarily driven by two genes, GTIIBS and Nkef. However, single‐gene Mantel tests comparing methylation and pairwise *F*_ST_ were not significant after Bonferroni correction. Thus, population differences in DNA methylation are more likely related to unmeasured oceanic environmental conditions, local adaptation, and/or genetic drift. DNA methylation is an additional mechanism that contributes to among population variation, with potential influences on organism phenotype, adaptive potential, and population resilience.

## Graphical abstract

DNA methylation presents a novel evolutionary mechanism for differences among populations due to its ability to respond to environmental change and its transmission among generations. We show population‐level differences in Chinook salmon DNA methylation and attempt to relate these differences to freshwater environmental context and genetic drift.

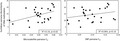

## 1 INTRODUCTION

Local adaptation occurs when organisms evolve in response to selective pressures in their immediate environment, resulting in increased individual fitness within their native habitat relative to non‐native habitats (García de Leániz et al., 2007; Kawecki & Ebert, 2004; Savolainen et al., 2013). Traditionally, the main mechanism considered to be underlying local adaptation has been genetic adaptation: selection acts upon the phenotypes produced by standing genetic variation, resulting in increased frequency of beneficial alleles and thus evolution of populations over multiple generations (Bernatchez, 2016). Additional mechanisms are now also accepted as contributing to local adaptation. Chromosomal translocations can result in co‐adapted gene complexes resistant to crossing‐over (Barth et al., 2019; Kess et al., 2020; Kirkpatrick & Barton, 2006; Lehnert et al., 2019). Maternal effects can influence offspring phenotype to prepare offspring for a predicted environment based on the experiences of the mother (Aykanat et al., 2012; Galloway, 2005; Galloway & Etterson, 2007). Phenotypic plasticity, whereby an organismal phenotype, is shifted toward an “ideal” phenotype based on the environment without underlying genetic changes (Hutchings, 2011; Pfennig et al., 2010; Torres‐Dowdall et al., 2012), which can be mediated by differences in gene expression (Fangue et al., 2006; Whitehead & Crawford, 2006; Wellband & Heath, 2013). These mechanisms can all occur simultaneously in an organism, leading to a wide variety of mechanistic contributions toward local adaptation.

Adaptive population differences in gene expression have been reported in a broad variety of taxa. Differences in gene expression occur among populations of killifish (*Fundulus heteroclitus*) across a natural thermal cline (Fangue et al., 2006), among rainbow trout (*O. mykiss*) from different tributaries subjected to stress challenges (Wellband & Heath, 2013), between populations of the copepod *Tigriopus californicus* residing in different thermal regimes (Schoville et al., 2012), among populations of *Drosophila subobscura* across latitudinal and thermal clines in Europe (Porcelli et al., 2016), and both within and among populations of teleost fish from the genus *Fundulus* (Oleksiak et al., 2002). Further, patterns in gene expression variation may also reflect parallel evolution due to similar environmental conditions (reviewed in Fraser et al., 2011). While local adaptation through gene expression variation has been frequently reported, the mechanisms underlying these differences in gene expression are poorly characterized, though environmental, genetic, and epigenetic variation could contribute to locally adapted gene expression profiles.

DNA methylation is one potential mechanism underlying transcriptional differences observed among populations in the context of local adaptation. DNA methylation is the addition of a methyl group to a cytosine (C) base that precedes a guanine (G) in the DNA sequence, known as a CpG site (Head, 2014). Numerous studies have shown that DNA methylation is highly sensitive to environmental signals (Barfield et al., 2014; Bossdorf et al., 2008; Foust et al., 2016; Herrera & Bazaga, 2010; Richards et al., 2010) and is involved in acclimation to environmental stress (Metzger & Schulte, 2017, 2018; Morán et al., 2013). Due to the potential to modify methylation in response to environmental cues, methylation presents an important mechanistic intersection between acclimation and adaptation, particularly with extensive evidence for rapid (or “contemporary”) evolution over short time scales (Stockwell et al., 2003). Methylation has been shown to be a highly targeted process (Venney et al., 2016, 2020). Therefore, short‐term changes in methylation can occur that allow an organism to cope with its environment, without the lag times associated with selection on standing genetic variation (Bossdorf et al., 2008; Hu & Barrett, 2017; Richards et al., 2010), consistent with rapid evolution. Due to the sensitivity of methylation to environmental cues, it presents a novel mechanism for organisms to adapt to their environment and adds an additional level of complexity in organismal phenotypic variation and evolution (Bossdorf et al., 2008). Furthermore, methylation may respond to environmental stress, allowing for targeted short‐term responses to environmental changes, which cannot occur through genetic adaptation (Hu & Barrett, 2017). If methylation results in phenotypic plasticity, it may act in lieu of genetic adaptation, since the detrimental phenotype is no longer present to be selected against, or it may prolong the persistence of organisms in stressful environments until selection and genetic adaptation can occur (Crispo, 2008).

Population‐level variation in methylation has been reported in a variety of species and appears to have an underlying genetic basis. While methylation can be taxon‐specific, studies in several taxa have identified a link between genetic and epigenetic variation (Fraser et al., 2012; Herrera & Bazaga, 2010; Liu et al., 2012). For example, a study in Spanish violets (*Viola cazorlensis*) across an elevation gradient identified a strong correlation between methylation and genetic variation using pairwise distance‐based AFLP analyses (Herrera & Bazaga, 2010). Similar results were found using restriction enzyme‐based methods for whole‐genome DNA methylation estimation and sequence polymorphism in female great roundleaf bat (*Hipposideros armiger*) populations (Liu et al., 2012), when comparing CpG‐specific methylation and sequence variation in oak (*Quercus lobata* Née) populations (Platt et al., 2015), and for correlations between methylation differences and allele frequencies among human ethnicities (Fraser et al., 2012). However, a study in salt marsh perennials (*Spartina alterniflora* **)** was unable to link genetic differences with variation in methylation through AFLP‐based approaches and instead found a strong correlation with environmental variation (Foust et al., 2016). Thus, the relationship among epigenetic variation, genetic variation, and environmental heterogeneity is unclear, yet characterizing the interactions between these three drivers of population‐level phenotypic variation is important in determining the role DNA methylation may play in driving local adaptation and thus population and species resiliency. While many studies have shown methylation differences among populations, most studies have focused on agriculturally important laboratory‐reared species, while studies of natural populations are limited (Richards et al., 2010), making the role of DNA methylation in population differentiation unclear.

Chinook salmon (*Oncorhynchus tshawytscha*) are a culturally, ecologically, and economically important species of Pacific salmon. There is ample evidence for local adaptation based on functional differences among populations of Chinook salmon resulting in increased fitness in their native environments (Fraser et al., 2011). Adaptive genetic variation occurs at selected immune and growth‐related candidate loci indicating genetic adaptation to their environment, while divergence at neutral (microsatellite) loci is related to isolation and genetic drift (Heath et al., 2006). Adaptation can occur within Chinook salmon stocks, for example, as evidenced by intrapopulation genetic differences in circadian clock genes based on migration timing, in the absence of neutral genetic variation (O'Malley et al., 2013). Variants impacting life‐history traits associated with environmental differences have also been reported in recently colonized Chinook salmon populations (Unwin et al., 2000), as well as differences in genetic variance components and fitness‐related traits (Aykanat et al., 2012). Thus, there is abundant evidence for adaptive differences among populations of Chinook salmon, though most studies focus on genetic differences. While there have been studies documenting neutral and functional genetic variation among populations of Chinook salmon, it is unclear how rapid adaptation occurs when local conditions change or salmon colonize new habitats. However, studies have shown evidence for rapid adaptation to hatchery rearing, resulting in differences in gene expression (Christie et al., 2016), reproductive success (Christie et al., 2012), and DNA methylation (Gavery et al., 2018; Le Luyer et al., 2017). Due to the role of DNA methylation in rapid evolution of salmonids, it is possible that DNA methylation is important for responding to environmental changes, as well as maintaining standing genetic variation in salmon.

The goal of this study is to determine the potential role of DNA methylation in maintaining differences (adaptive or drift‐related) among populations and to assess genetic and environmental drivers of population‐level differences in methylation. We characterize locus‐specific population differences in DNA methylation in Chinook salmon while testing for the effects of developmental timing and sampling year. We associate population differences in methylation with freshwater environmental differences and genetic drift on levels of methylation at selected genes. We obtained eyed eggs from eight populations of Chinook salmon and measured DNA methylation using a gene‐targeted PCR‐based DNA methylation assay for next‐generation sequencing. We expected that populations would exhibit different levels of DNA methylation at specific functional loci. Such patterns of methylation differences among populations could be due to environmental acclimation (Foust et al., 2016), underlying adaptive genetic variation (Fraser et al., 2012; Herrera & Bazaga, 2010; Liu et al., 2012), or maternal effects at the eyed egg stage (Venney et al., 2020). These differences would likely show developmental and interannual variation due to strict developmental control of DNA methylation and interannual environmental variation. We hypothesized that population differences in methylation should occur in specific genes in response to unique environmental conditions and/or selective pressures among natural environments. We tested for correlations between locus‐specific methylation and freshwater environmental variables from the native rivers of each population to determine whether local environmental factors influence gene‐specific DNA methylation differences. We also tested for a correlation between genetic drift (variation at neutral marker loci) and methylation differences among populations to determine whether methylation differences could be explained by population divergence due to genetic drift (and/or geographic isolation) distance. DNA methylation presents an additional, potentially powerful and rapid mechanism through which populations can respond to their environments, cope with environmental stress, and evolve.

## 2 MATERIAL AND METHODS

### 2.1 Eyed egg sampling and DNA extraction

**FIGURE 1:**
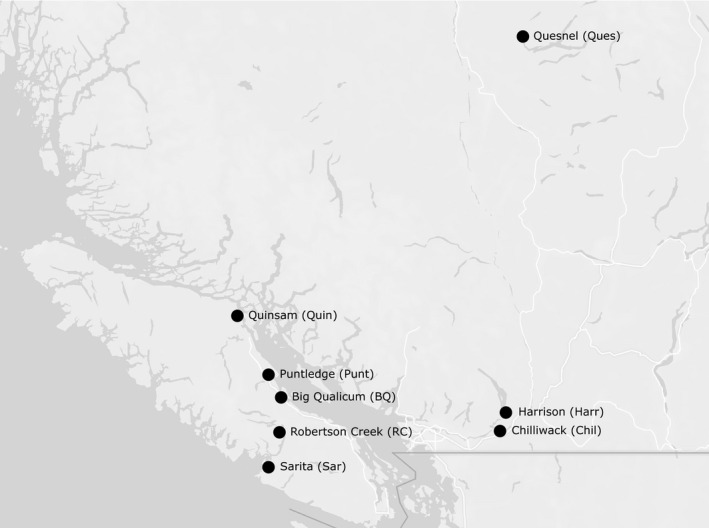
Locations of source populations of Chinook salmon eyed eggs sampled from DFO hatcheries in 2015. Eggs were obtained from Big Qualicum Hatchery (BQ), Chilliwack River Hatchery (Chil), Chehalis River Hatchery (Harr), Puntledge River Hatchery (Punt), Quinsam River Hatchery (Quin), Robertson Creek Hatchery (RC), and Nitinat River Hatchery (Sar). Quesnel River eggs were obtained from another project

Sampling adhered to Canadian Animal Care guidelines as approved by the University of Windsor (ACC #17‐08). Eyed eggs (embryos) were sampled from eight populations of Chinook salmon from bulk incubators containing offspring from multiple mothers. The age of eggs, which did not vary within a population, ranged from 323 to 445 accumulated temperature units (ATUs). Samples from seven populations were obtained from DFO Salmon Enhancement Program hatcheries in November 2015 by hatchery staff while Quesnel River eggs were obtained from another project (Figure 1). Additional samples were obtained from Big Qualicum (BQ) and Harrison (Harr) populations in 2017 to test for interannual variation in methylation. Eggs were immediately preserved in a high salt buffer (25 mM sodium citrate, 10 mM EDTA, 5.3 M ammonium sulfate, pH 5.2) for future analysis.

Embryos were dissected from 48 eyed eggs per population (*n* = 10). The yolk was removed and the embryos were digested whole in 10 µl of 20 mg/ml proteinase K and 1,000 µl of digestion buffer (100 mM NaCl, 50 mM Tris‐HCl pH 8.0, 10 mM EDTA, 0.5% SDS) at 37°C for 24 hr. We used 150 µl of the digested product for DNA extraction in a high‐throughput automated plate‐based DNA extraction protocol (Venney et al., 2020).

### 2.2 Bisulfite conversion and sequencing

DNA concentration was determined using a Quant‐IT PicoGreen® dsDNA Assay Kit. Approximately 500 ng of DNA underwent bisulfite conversion using a Zymo EZ‐96 DNA Methylation‐Lightning kit following the manufacturer's protocol. Bisulfite conversion converts unmethylated cytosines to uracil while not affecting methylated cytosines, allowing for the determination of sites of methylation in the DNA sequence.

Methylation analysis was performed with 21 published bisulfite sequencing primers (Venney et al., 2016) and two novel bisulfite sequencing primer sets for *growth hormone 2* (GH2, forward primer 5′‐TTATTAAACCTTTCTAAAAACACAC‐3′, reverse primer 5′‐ATTTAAATTTTAATTTTTTATAGGG‐3′, 241 bp fragment excluding primer sequences) and *heat shock factor 1b* (hsf1*b*, forward primer 5′‐AGGATTAGGATTTTGAAGAGGATTT‐3′, reverse primer 5′‐AATTAATTTTTCATCATCTACACATTAACA‐3′, 132 bp fragment excluding primer sequences). All primers were designed for gene regions with little to no sequence variation to minimize the effects of genetic variation on the interpretation of DNA methylation data. Assayed genes were selected for their roles in early development, stress and immune function, metabolism, early growth, and differentiation. Amplicons ranged from 79 to 249 bp, with a total of 4,111 bp sequenced excluding primer sequences (Table 1). PCRs were performed using a two‐stage PCR approach (Venney et al., 2016) where the first stage amplified the targeted gene region, and the second stage ligated sample barcode and adaptor sequences to the amplicon. Barcode sequences are 10–12 bp unique sequences that allow for the identification of individual samples in massively parallel (next‐generation) sequencing. Samples were split among three sequencing runs and sequenced with an Ion 318™ Chip using an Ion PGM™ Sequencing 400 bp kit on the Ion Torrent Personal Genome Machine® (PGM™) with an expected 500 reads per gene per sample.

**TABLE 1:** Bisulfite sequencing primer sequences for Chinook salmon

Gene	Forward and reverse primer sequences (5′ to 3′)
Growth genes	
Brain‐derived neurotrophic factor (BDNF)	GATTAAGGATGTTGATTTGT
	TAACAATCTACCCAAACATATCTAT
Follicle‐stimulating hormone beta (FSHb)	TGTGTAATTTTAAGGAGTGGTTTTA
	ACATTTCTAATAAATTTACTATACAACTAA
Growth hormone 1 (GH1)	TTTAGTTAGAAAGTATAGTGTAAGGATTA
	TTATTAAACCTTTCTAAAAACACAC
Growth hormone 2 (GH2)	ATTTAAATTTTAATTTTTTATAGGG
	CAATCAATAAAATAAATTACCCCATCAC
Gonadotropin II beta subunit (GTIIBS)	TTTTGTGTATTTATTTATTAGGAGT
	ATACAAAAATCTAACTACAAACTCTC
Pituitary‐specific transcription factor (pit1)	GAGAATTTGTAGTTGAGTTTTAAGA
	AAAATAAAAACTTAATCTTCTCCCC
Immune‐ and stress‐related genes	
Antithrombin (anthr)	TTAAATATTTTTATGTTTTTTATTA
	TCTCAATCTTAATTTTATATTTT
Chemokine 1 (CK1)	TTTTTTTTTTTTATTATTATTTTTA
	CTAAATAAACTTCAAACAACAATC
Heterogeneous nuclear ribonucleoprotein L (hnrL)	TATATTTGAGTTTAATTTTGGAAT
	CACACCATTTAAATAAAACCATAAT
Interleukin 8 receptor (IL8R)	TTTGTTTTTATTATTTATTATGGTGG
	AAATACACCAACTTAACCCTCATC
Natural killer enhancement factor (Nkef)	TAGAATAATATTTTTAGTATTTTTT
	TTCCTCATTTCAAACTATCCCATCT
Recombination activating gene 1 (RAG1)	TTTAAGTTTAATTTAGAGATGTTTT
	CCTCCAAACCCTCCATCTCTCACAC
Transferrin (Tf)	ATAGTATTTATTTTGTTTTTAGTTA
	CTCACCTTAATAACTTTAATACATTCAAAA
Metabolic genes	
Heat shock cognate protein 71 (hsc71)	TTGATTTTGGTTTAATTATTTGAGGA
	TCAAACACTCCCTAATACCATTTAC
Heat shock factor 1b (hsf1b)	AGGATTAGGATTTTGAAGAGGATTT
	AATTAATTTTTCATCATCTACACATTAACA
Heat shock protein 47 (hsp47)	AAGTATTTTTAGGGAATAGGAGTGTATATA
	TATCTAATTTTATAAAAAACAAAAATCAAA
Heat shock protein 70 (hsp70)	TAGTTGTTAAGAATTTTTTGGAGT
	AACTAATACTCATACTCCTCTTTATC
Heat shock protein 70a (hsp70a)	GTAGGGAAATTTTTGTTTTATTG
	CCAATTATTTTAATAACTACTATCTTATCT
Heat shock protein 90 (hsp90)	ATGAGATTTTATTTTTAGAGGGAGA
	CCATAAAAAACACTAACCAAATTACC
Inosine triphosphatase (itpa)	TTGTGTAGATTAGATAGTTTTATAT
	AATCCAAATTTAATAAACTCTATCAATTTA
Metallothionein A (metA)	TTTATGGTAAATTTAATTAATTTTAATTGT
	AACCTAAAACACACTTACTACAACC
Myosin 1A (Myo1A)	TGTAGGAGTTAGTTTTTGGTAAAGTAT
	AAAAATCAATCTAAACTCACCAATC
Tumor antigen P53 (P53)	GGTTTTGGGTTGATTTTTAATTAAT
	ATTAATCTCCTCTATCTTCCTATCTC

### 2.3 Bisulfite sequencing data processing

Sequence data files were demultiplexed using *mothur* (Schloss et al., 2009), primer sequences were trimmed, and one fastq sequence file was created per individual. Bisulfite sequence data were aligned with known genomic sequences using *bwa‐meth* (Pedersen et al., 2014) with a maximum of two mismatches per sequence to ensure sequences represented the target genes. Tabulated methylation data from *bwa‐meth* were imported into R (R Development Core Team, 2021) for quality filtering to ensure the same CpG sites were compared across all samples: CpG sites were excluded from the analysis if they were sequenced (a) with fewer than five reads per gene per sample (though generally greatly exceeded this requirement) and (b) in less than 70% of individuals. Rosner's test for extreme outliers was used to exclude significant outlier data points, which were likely reflections of low sequence depth rather than biologically meaningful variation. The final processed data provided average percent methylation for each individual in each gene that surpassed quality guidelines. Average methylation levels are often a good predictor of gene transcription (Gavery & Roberts, 2013; Wagner et al., 2014), though this effect varies based on the gene region studied (Anastasiadi et al., 2018; Wagner et al., 2014).

### 2.4 Sampling year and ATU effects on methylation

Due to differences in ATUs (accumulated temperature units, a measure of developmental timing in salmon) among populations, and within populations among sampling years, we tested for ATU effects on gene‐specific methylation since developmental stage can have significant effects on methylation. Using the average percent methylation data, we determined the median methylation percentage for each gene in each population and used a linear regression per gene using the per population median methylation percentage to test for the effect of ATU on median methylation levels. We corrected for multiple comparisons using a Benjamini–Hochberg false discovery rate (FDR) correction. As developmental stage was found to be correlated with methylation (see Results), we used the residuals from linear models of ATU effects on single‐gene methylation instead of raw methylation data for all analyses to control for the effect of ATU on methylation.

We tested for the effect of sampling year on methylation using residuals generated from linear regressions for 20 loci for the BQ and Harr 2015 and 2017 samples. For this analysis, we used only 20 loci due to three loci being excluded by quality filtering. An ANOVA was used for each gene to test for the effects of population, year, and their interaction using only BQ and Harr gene methylation data to determine whether methylation changed from year to year. *p*‐values were corrected using a Benjamini–Hochberg false discovery rate (FDR) correction.

### 2.5 Population effects on methylation

We tested for population‐level effects across all genes using the 2015 samples (8 populations) to determine whether overall methylation differs among populations using an ANOVA for the effects of population, gene, and their interaction. An FDR‐corrected ANOVA was used to test for the effect of population on individual gene methylation variation to determine which genes were driving population differences in methylation. Tukey's HSD post hoc test in the R package *agricolae* v1.3.2 (de Mendiburu, 2020) was used to determine whether specific populations showed consistently different methylation levels across genes. R^2^ values were obtained from all ANOVAs to estimate the methylation variance explained among populations, both across all genes, and for individual gene loci.

### 2.6 Principal component regressions for environmental effects on DNA methylation

To determine whether environmental variation was driving population‐level differences in methylation, we gathered data for 23 environmental variables from each natal river. In addition to longitude and latitude for each location, average temperature and precipitation were tabulated from the Government of Canada's historical climate database for the nearest available region (available at https://climate.weather.gc.ca/). The Government of British Columbia's iMapBC app (https://www2.gov.bc.ca/gov/content/data/geographic‐data‐services/web‐based‐mapping/imapbc) was used to determine water turbidity, as well as concentrations of nitrite, nitrite + nitrate, chloride, and 14 metals in each river using water quality monitoring data (Table 2). Where possible, mean environmental data from several nearby monitoring stations were used. An estimate of pathogen diversity based on the number of diseases reported for fish from each population was included from the Government of Canada's Fish Health Database (https://open.canada.ca/data/en/dataset/2ece9991‐62aa‐4b7a‐bd7d‐4f8f1052cd21).

**TABLE 2:** Climate (https://climate.weather.gc.ca/) and water quality data (https://www2.gov.bc.ca/gov/content/data/geographic‐data‐services/web‐based‐mapping/imapbc) for natal streams of eight populations of Chinook salmon

Population	BQ	Chil	Harr	Punt	Ques	Quin	RC	Sar
Latitude	49.393902	49.08082	49.27145	49.68617	52.65973	50.01665	49.33967	48.89538
Longitude	−124.618084	−121.704959	−121.91462	−125.03228	−121.69789	−125.30218	−124.98791	−124.96138
Average Temperature (°C, September–November)	9.47	9.57	11.00	9.43	4.87	9.40	9.90	10.13
Precipitation (mm, September–November)	411.60	477.60	564.30	431.10	157.00	497.70	634.40	869.30
Turbidity (NTU)	0.59	4.59	2.15	1.06	1.32	1.35	0.29	0.60
Al (mg/L)	0.05	0.00	0.63	0.08	0.04	0.05	0.06	0.12
As (mg/L)	0.25	0.00	0.06	0.05	0.00	0.09	0.04	0.00
Ca (mg/L)	10.13	26.68	11.10	5.91	16.88	13.39	4.95	2.79
Cd (mg/L)	0.01	0.00	0.00	0.00	0.00	0.00	0.00	0.00
Co (mg/L)	0.10	0.00	0.01	0.04	0.00	0.03	0.00	0.00
Cr (mg/L)	0.01	0.00	0.00	0.00	0.00	0.00	0.00	0.00
Cu (mg/L)	0.01	0.02	0.00	0.00	0.00	0.00	0.00	0.00
Fe (mg/L)	0.10	0.02	0.31	0.17	0.05	0.14	0.05	0.07
Mg (mg/L)	1.78	5.48	3.10	1.20	1.91	3.04	0.39	0.56
Mn (mg/L)	0.01	0.00	0.01	0.01	0.00	0.01	0.00	0.01
Mo (mg/L)	0.01	0.00	0.01	0.00	0.00	0.00	0.00	0.00
Ni (mg/L)	0.05	0.00	0.02	0.02	0.00	0.01	0.01	0.00
Pb (mg/L)	0.10	0.00	0.03	0.02	0.00	0.03	0.00	0.00
Zn (mg/L)	0.01	0.01	0.00	0.00	0.00	0.00	0.01	0.00
Chloride (mg/L)	5.00	0.50	0.20	1.60	0.51	2.20	0.87	3.80
Nitrate + Nitrite (mg/L)	0.05	0.06	0.07	0.06	0.10	0.13	0.02	0.04
Nitrite (mg/L)	0.01	0.00	0.01	0.00	0.00	0.01	0.01	0.00

Due to the large number of environmental variables collected, a principal component analysis (PCA) was used to reduce the dimensionality and autocorrelation of the environmental dataset. Principal components (PCs) were retained based on examination of a Scree plot and the eigenvalues of the PCs exceeding 1.0. To determine the effect of environmental factors on population differences in locus‐specific methylation, a linear model was used to test the effects of each individual PC on methylation at each locus with a significant population effect on methylation (i.e., one linear model per PC per gene to avoid overfitting models for a small sample size). For all PC regressions, population medians from the residuals of ATU regressions on methylation were used instead of raw methylation data to minimize pseudoreplication and to control for the confounding effects of ATU. For each PC, a linear model was used to determine the effect of the PC on population‐level differences in single‐gene methylation, and an FDR correction was used to correct for multiple comparisons.

### 2.7 Mantel tests comparing methylation data to microsatellite and SNP pairwise *F*_ST_

Since the methylation assay targets conserved regions of the target genes, measures of genetic differentiation between the studied populations were obtained from existing databases at the Molecular Genetics Lab (Fisheries and Oceans Canada). Selected populations from the genetic baseline for Chinook salmon amplified by a microsatellite panel with 15 markers (similar to Beacham et al., 2006) or a SNP panel with a minimum of 195 markers per sample and maximum of 369 markers (Beacham et al., 2018) were exported in genepop format. The microsatellite data serve as an estimate of neutral genetic drift among the populations. The SNP panel represents highly divergent (likely adaptive and neutral) SNPs among populations and serves as an additional estimate of genetic divergence. The SNP data specifically aimed to use fall populations when possible (i.e., Harr, Puntledge, and Chilliwack River). The Chilliwack population was restricted to the 2018 brood year. These datasets were analyzed using custom R scripts (R Development Core Team, 2021). In brief, datasets were loaded into R using adegenet v.2.1.1 (Jombart, 2008), and dendrograms were constructed using the aboot function of poppr v.2.8.3 (Kamvar et al., 2014) with the edwards.dist metric (Cavalli‐Sforza & Edwards, 1967) using 10,000 bootstraps. Data were then converted from genind format to hierfstat format using the genind2hierfstat function of hierfstat v.0.04‐22 (Goudet, 2005), and then, pairwise *F*_ST_ values were calculated using the pairwise. WCfst (Weir & Cockerham, 1984) function within hierfstat.

Pairwise distance matrices for microsatellite and SNP data were compared to methylation matrices to determine whether population‐level differences in methylation corresponded with expected divergence due to isolation and genetic drift. A Euclidean distance matrix for population‐level methylation variation was generated in the R package *ade4* (Dray & Dufour, 2007) using the medians of the residual methylation data across the eight genes showing significant population effects. The methylation distance matrix was compared to the pairwise microsatellite and SNP *F*_ST_ matrices using Mantel tests with 99 permutations in GenAlEx (Peakall & Smouse, 2006, 2012) to determine whether population differences in methylation across the eight genes were consistent with genetic divergence. A Euclidean distance matrix was generated for the median residual data of the ATU‐corrected methylation profile for each gene to determine whether population differences in single‐gene methylation fit with genetic drift expectations (i.e., neutral genetic distance correlated with methylation difference). We used a Bonferroni‐corrected Mantel test with 99 permutations to determine whether divergence in methylation for each of the eight gene loci showing significant population effects corresponded with population‐level genetic variation assessed by either microsatellite or SNP variation (*F*_ST_).

## 3 RESULTS

### 3.1 Effects of ATU and sampling year on methylation profiles

Average coverage across all CpGs included in the analysis for a given gene was 222 reads (range = 44–514). Linear regression results showed that accumulated temperature unit (ATU) significantly affected *chemokine 1* (CK1) methylation before FDR correction (*p* = .02, *p* = .44 after FDR, adjusted *R* ^2^ = 0.56) and approached statistical significance for four other loci: *follicle‐stimulating hormone* (FSHb), *growth hormone 1* (GH1), *heat shock protein 90* (hsp90), and *metallothionein A* (metA); .1 > *p* > .05 before FDR correction). Since DNA methylation is significantly affected by developmental age (Anastasiadi & Piferrer, 2020; Mayne et al., 2020) and our results support age effects on locus‐specific methylation, we controlled for the effect of developmental timing (ATU) on methylation. Thus, residuals from the linear regression for the effects of ATU on gene‐specific methylation for all 48 individuals per population were used instead of raw methylation data to control for the potentially confounding effects of developmental timing.

We found no significant year effects on ATU‐corrected methylation (after FDR correction) for the 2015 and 2017 BQ and Harr samples. We did, however, find population effects on methylation across years for BQ and Harr (2015 and 2017 samples) for *gonadotropin II beta subunit* (GTIIBS, *p* < .01), *natural killer enhancement factor* (Nkef, *p* < .001), and hsp90 and CK1 (*p* < .05) after FDR correction (Table 3). We identified a population by year interaction effect on Nkef methylation (*p* < .01 after FDR correction). Due to the Nkef population by year effect, as well as other significant interaction effects (before FDR correction), only residuals from ATU models for the 2015 samples were used for downstream statistical analyses to avoid potential annual variances in methylation. However, population and the population by year interaction contributed considerably more to variation in methylation than sampling year (Table 3).

**TABLE 3:** ANOVA results for the effects of population, year, and population by year interaction on methylation residuals from ATU regressions for 20 genes in Chinook salmon

Gene	*p*‐value (FDR corrected)			Mean squares						Percent phenotypic variance			
	Population	Year	Population × Year	Population	Year	Population × Year	Residual	Total		Population	Year	Population × Year	Residual
FSHb	0.998	0.998	0.960	1.8	0.0	3.8	3.8		9.4	19.3	0.3	40.1	40.3
GTIIBS	***0.002***	0.998	0.560	346.1	0.2	46.4	21.1		413.8	83.6	0.0	11.2	5.1
GH1	0.998	0.998	0.998	6.7	0.0	0.2	13.3		20.1	33.1	0.0	0.9	66.0
GH2	0.960	0.998	0.865	35.2	1.1	46.0	33.9		116.1	30.3	0.9	39.6	29.2
hsf1b	0.998	0.998	0.151	2.7	1.6	217.1	41.1		262.4	1.0	0.6	82.7	15.7
hsp90	***0.048***	0.998	0.986	123.6	0.0	12.8	14.3		150.7	82.0	0.0	8.5	9.5
metA	0.998	0.998	0.998	1.0	0.0	0.0	2.6		3.7	28.3	0.0	0.1	71.6
pit1	0.998	0.998	0.998	0.0	0.0	0.3	8.0		8.4	0.5	0.0	3.4	96.1
IL8R	0.957	0.998	0.998	3.7	0.0	0.8	3.3		7.8	47.8	0.2	10.1	41.9
Tf	0.998	0.998	0.998	0.1	0.0	0.0	0.2		0.3	20.2	0.0	0.1	79.7
p53	0.077	0.998	0.077	79.1	0.3	79.3	11.4		170.1	46.5	0.2	46.6	6.7
hsc71	0.998	0.998	0.160	147.6	8.3	945.6	189.1		1,290.6	11.4	0.6	73.3	14.7
hsp47	0.998	0.998	0.104	12.4	1.6	195.6	31.7		241.3	5.1	0.7	81.1	13.1
hsp70a	0.998	0.998	0.998	0.2	0.0	0.1	5.7		5.9	3.5	0.0	1.3	95.2
CK1	***0.048***	0.998	0.803	146.2	1.2	26.6	17.1		191.1	76.5	0.6	13.9	8.9
ITPA	0.252	0.998	0.424	182.8	0.5	136.3	45.4		365.0	50.1	0.1	37.3	12.4
BDNF	0.560	0.998	0.998	10.9	0.0	0.0	4.8		15.8	69.2	0.0	0.3	30.5
hnrL	0.998	0.998	0.560	0.3	0.1	12.4	5.3		18.1	1.9	0.5	68.5	29.1
anthr	0.998	0.998	0.998	0.1	0.0	1.6	7.8		9.6	0.7	0.1	17.2	82.0
Nkef	***0.000***	0.998	***0.001***	2,951.3	8.7	1,966.7	110.1		5,036.8	58.6	0.2	39.0	2.2
								Average		33.5	0.3	28.8	37.5

Note

Fish were sampled from Big Qualicum and Harrison River in 2015 and 2017 to test for an interannual effect on methylation. Presented are (a) FDR‐corrected *p*‐values, (b) mean square estimates, and (c) percent phenotypic variance attributed to each term. Significant *p*‐values are bolded and italicized.

### 3.2 Population differences in methylation

Population and the population by gene interaction significantly affected methylation levels across all genes combined (both *p* < .001, *R* ^2^ = 0.10), indicating that while populations differ in overall methylation levels, they also differ in extents of gene‐specific methylation. Direct between‐gene differences in methylation were not quantifiable, as gene methylation values were standardized and centered around zero by using the ATU model residuals (*p* = 1.0).

DNA methylation differed among populations for eight genes: three heat shock proteins (all *p* < .01 after FDR correction) including *heat shock protein 70* (hsp70), hsp90, and *heat shock protein 47* (hsp47); GTIIBS, *tumor suppressor protein 53* (p53), *heat shock cognate 71* (hsc71), and *recombination activating gene 1* (RAG1), and Nkef (all *p* < .001 after FDR correction, Table 4 for *p*‐values and *R* ^2^ values). Tukey's HSD post hoc test identified similarities in Nkef, RAG1, and p53 methylation levels among BQ, Puntledge (Punt), Quinsam (Quin), and Sarita (Sar), though no other patterns are apparent.

**TABLE 4:** *p*‐values and *R* ^2^ values from ANOVAs and Mantel tests for population effects on DNA methylation in Chinook salmon

Gene	ANOVA for population effect		Mantel test for correlation with microsatellite *F*_ST_		Mantel test for correlation with SNP *F*_ST_	
	*p*‐value (FDR correction)	Adjusted *R* ^2^	*p*‐value	Adjusted *R* ^2^	*p*‐value	Adjusted *R* ^2^
FSHb	.646	−0.003				
GTIIBS	.***000***	0.192	.020	0.245	.254	0.010
GH1	.429	0.005				
GH2	.799	−0.009				
hsf1b	.175	0.016				
hsp70	.***003***	0.048	.100	0.102	.2522	0.050
hsp90	.***005***	0.042	.330	0.009	.0199	0.350
metA	.450	0.003				
pit1	.320	0.008				
IL8R	.646	−0.002				
Tf	.200	0.014				
p53	.***000***	0.091	.310	0.027	.0053	0.300
Myo1A	.263	0.011				
hsc71	.***000***	0.063	.380	0.000	.0237	0.300
hsp47	.***003***	0.047	.150	0.058	.0537	0.170
hsp70a	.646	−0.003				
RAG1	.***000***	0.172	.190	0.047	.0015	0.570
CK1	.066	0.024				
ITPA	.287	0.010				
BDNF	.786	−0.008				
hnrL	.767	−0.006				
anthr	.646	−0.003				
Nkef	.***000***	0.227	.030	0.201	.106	0.010

Note

ANOVAs tested for significant population effects on methylation. Mantel tests tested for a correlation between a Euclidian distance matrix for DNA methylation and both microsatellite and SNP pairwise *F*_ST_ divergence to determine whether differences in DNA methylation among populations were explained by genetic differentiation. Significant *p*‐values are bolded and italicized.

### 3.3 Principal component regressions for environmental effects on methylation

Six principal components explained 98.9% of variation in the environmental dataset. These PCs were retained in the analysis based on PC eigenvalue >1 and the relative values of PCs in the Scree plot (Table 5). The results of the principal component regression analysis showed that no environmental PC was significantly associated with population‐level methylation at any of the eight gene loci that differed among populations.

**TABLE 5:** PCA loadings for 23 environmental variables gathered for natal streams of eight Chinook salmon populations

Variable	PC1	PC2	PC3	PC4	PC5	PC6
Latitude	0.112	0.035	−0.258	0.440	−0.163	0.074
Longitude	0.200	0.314	0.065	0.091	−0.134	0.302
Pathogen diversity	−0.017	0.117	0.407	0.240	0.042	0.274
Average Temperature (°C, September–November)	−0.129	−0.014	0.342	−0.342	0.088	−0.190
Precipitation (mm, September‐November)	−0.032	−0.256	0.308	−0.275	0.222	−0.025
Turbidity (NTU)	0.196	0.335	0.047	−0.188	0.175	−0.027
Al (mg/L)	−0.037	0.129	0.459	0.140	−0.187	0.053
As (mg/L)	−0.318	0.141	−0.115	−0.019	0.011	0.001
Ca (mg/L)	0.176	0.365	−0.156	−0.058	0.126	−0.038
Cd (mg/L)	−0.313	0.137	−0.069	−0.055	−0.120	0.177
Co (mg/L)	−0.308	0.111	−0.184	−0.015	0.083	0.033
Cr (mg/L)	−0.318	0.150	−0.099	0.019	−0.079	0.036
Cu (mg/L)	0.077	0.342	−0.072	−0.344	0.156	0.027
Fe (mg/L)	−0.120	0.148	0.386	0.204	−0.111	−0.161
Mg (mg/L)	0.127	0.395	0.015	−0.117	0.224	−0.155
Mn (mg/L)	−0.240	−0.149	0.035	0.193	0.417	0.104
Mo (mg/L)	−0.251	0.251	0.168	−0.033	−0.222	−0.017
Ni (mg/L)	−0.322	0.153	−0.043	−0.014	−0.086	0.051
Pb (mg/L)	−0.310	0.179	−0.083	0.022	0.036	0.083
Zn (mg/L)	−0.050	−0.084	−0.192	−0.340	−0.490	−0.165
Chloride (mg/L)	−0.254	−0.125	−0.111	−0.058	0.392	0.218
Nitrate + Nitrite (mg/L)	0.035	0.166	−0.101	0.372	0.271	−0.438
Nitrite (mg/L)	−0.195	−0.005	0.037	0.126	−0.037	−0.644

### 3.4 Mantel tests comparing methylation data to genetic differentiation (*F*_ST_)

**FIGURE 2:**
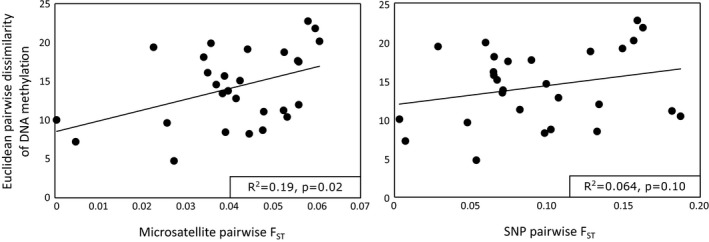
Scatter plots of pairwise Euclidean dissimilarity matrix for residual methylation medians (eight genes) versus (a) microsatellite *F*_ST_ values based on data from 15 loci, and (b) SNP *F*_ST_ values based on up to 389 loci. The solid lines (and boxed statistics) show results of Mantel tests for correlation

Microsatellite pairwise *F*_ST_ values ranged from 0.00041 to 0.061 while SNP pairwise *F*_ST_ values ranged from 0.0032 to 0.19 (Table 6). Pairwise Euclidean dissimilarity values for methylation data ranged from 4.76 to 22.7 (Table 7). The Mantel test (Table 4) comparing microsatellite pairwise *F*_ST_ to median residual methylation data for all eight genes (combined) with a significant population effect showed a weak correlation between population‐level differences in methylation and microsatellite genetic divergence (*p* = .02, *R* ^2^ = 0.19, Figure 2), suggesting weak effects of genetic drift on methylation. The Mantel test comparing SNP pairwise *F*_ST_ to methylation data across all eight genes was not significant (*p* = .10, *R* ^2^ = 0.064). Mantel tests correlating pairwise *F*_ST_ values with median residual methylation data for each gene were not significant.

**TABLE 6:** Pairwise *F*_ST_ estimates for SNP (above diagonal) and microsatellite (below parallel) markers estimating divergence among populations of Chinook salmon

	BQ	Chil	Harr	Punt	Ques	Quin	RC	Sar
BQ		0.071	0.065	0.003	0.163	0.054	0.065	0.089
Chil	0.040		0.007	0.071	0.187	0.107	0.102	0.134
Harr	0.035	0.005		0.065	0.182	0.099	0.098	0.128
Punt	0.000	0.038	0.034		0.159	0.048	0.060	0.082
Ques	0.060	0.053	0.048	0.058		0.157	0.132	0.149
Quin	0.027	0.041	0.037	0.026	0.061		0.067	0.074
RC	0.039	0.048	0.045	0.036	0.039	0.042		0.029
Sar	0.056	0.056	0.053	0.052	0.044	0.056	0.023	

**TABLE 7:** Pairwise Euclidean dissimilarity matrix for population‐level differences in methylation data across eight genes showing a significant population effect on methylation

	BQ	Chil	Harr	Punt	Ques	Quin	RC	Sar
BQ								
Chil	13.76							
Harr	16.12	7.23						
Punt	10.03	13.44	18.09					
Ques	21.81	10.41	11.09	22.74				
Quin	4.76	12.80	14.58	9.63	20.15			
RC	15.68	8.72	8.25	19.89	8.45	15.08		
Sar	17.65	11.96	18.74	11.25	19.12	17.49	19.40	

## 4 DISCUSSION

DNA methylation presents an evolutionary mechanism for individuals to rapidly respond to environmental changes and improve their survival in natural systems. In contrast, novel beneficial genetic mutations and natural selection acting upon existing variation can be slow processes that take place over generations (Bossdorf et al., 2008; Hu & Barrett, 2017; Richards et al., 2010). Previous epigenetic studies have primarily focused on sources of individual variation, rather than population‐level differences in methylation (Hu & Barrett, 2017), yet population‐level differences in methylation could explain heritable variation among populations which cannot be explained solely by genetic variation (Bossdorf et al., 2008). We observed significant population differences in methylation across all genes combined, as well as a significant population by gene interaction, indicating that populations differ in overall methylation, and also that the extent of methylation differences between populations varies among individual genes. Methylation differences among populations have been reported in several other studies (Barfield et al., 2014; Foust et al., 2016; Fraser et al., 2012; Herrera & Bazaga, 2010; Liu et al., 2012; Platt et al., 2015; Richards et al., 2010) with the potential to contribute to rapid acclimation and/or adaptation to stressors (Bossdorf et al., 2008; Hu & Barrett, 2017; Richards et al., 2010). The population‐level differences in methylation we report may represent an important evolutionary mechanism that could contribute to the extensive adaptive variation observed in natural populations of Chinook salmon (Fraser et al., 2011). However, the patterns of considerable population‐level variation in DNA methylation reported here are of broad interest when considering potential mechanisms of phenotypic differentiation in natural populations in general.

Population‐level differences in methylation could reflect short‐term acclimation to the local environment, or local adaptation due to environmental selection on heritable phenotypes. While several studies have identified population differences in methylation, most focus on methylation at the genome‐wide or whole‐genome level rather than using a candidate gene approach. We observed population‐level differences in methylation at specific genes in Chinook salmon eyed eggs: four heat shock protein genes (hsc71, hsp47, hsp70, and hsp90), three immune genes (p53, RAG1, and Nkef), and one gene involved in endocrine function (GTIIBS), all of which are logical targets for differences in methylation among populations. Heat shock proteins have a variety of cellular roles and become upregulated in stressed organisms in response to a broad variety of stressors and environmental situations, often with clinal or population‐level differences in heat shock protein expression (Sørensen et al., 2003; Tine et al., 2010). Previous studies in teleost fish have identified differences in immune response among populations (Evans et al., 1997, 2010; Fraser et al., 2011), as well as differences in hormone concentrations and endocrine function (Carr & Patiño, 2011; Sopinka et al., 2017). Differences in gene methylation could reflect acclimation or adaptation to local environments, though further research is required to determine whether population‐level differences in gene‐specific methylation result from acclimation or adaptation. However, significant differences in methylation between BQ and Harr with no significant temporal effects suggest local adaptation. Future research measuring methylation in reciprocal transplants or in common garden experiments with natural populations could determine whether population‐level variation in methylation is retained and therefore whether it likely represents acclimation or adaptation. Regardless of the underlying process, the genes showing significant population effects are logical targets for differential DNA methylation due to differences in environmental context and stressors among populations.

DNA methylation is often influenced by environmental context (Barfield et al., 2014; Bossdorf et al., 2008; Foust et al., 2016; Herrera & Bazaga, 2010; Richards et al., 2010). We used principal component analysis and regression to test for environmental effects on DNA methylation among populations using environmental data from the natal streams of the studied Chinook salmon populations. We found no significant effects after correcting for multiple comparisons, which was unexpected, as many studies have reported environmental effects on methylation (Angers et al., 2010; Dimond & Roberts, 2016; Foust et al., 2016; Le Luyer et al., 2017; Morán et al., 2013). The lack of significant environmental correlates is likely due to our use of Chinook salmon eggs. At the egg stage, the embryo is isolated and protected from the environment, which may reduce its response to environmental variation, though it is still possible that eggs respond to local environmental conditions through changes in methylation. If methylation in eggs is genetically encoded rather than environmentally determined, these differences in methylation may reflect genetic adaptation rather than acclimation. Alternatively, Chinook salmon exhibit strong maternal effects on DNA methylation at the eyed egg stage (Venney et al., 2020) which may increase variation within a population and reduce correlations between gene‐specific DNA methylation and environmental variables. Parents experience the freshwater environment prior to spawning and therefore could alter egg methylation signals in response to the offspring's predicted environment. Thus, the population‐level differences in methylation observed in Chinook salmon may be due to acclimation or adaptation to freshwater environmental signals from their parents, eyed egg acclimation to the environment, or due to genetic differences among populations (Fraser et al., 2012; Liu et al., 2012).

Population epigenetic studies in different taxa vary in their conclusions as to the link between epigenetic differences among populations and genetic divergence. A study in salt marsh perennial plants found no link between genetic and epigenetic differences across environmental gradients, but a strong correlation with environmental conditions (Foust et al., 2016). However, another study linked DNA methylation differences in Spanish violets to genetic differences identified by AFLP in response to elevation (Herrera & Bazaga, 2010). A significant correlation between genetic and epigenetic variation was also reported among female great roundleaf bat populations (Liu et al., 2012) and due to differences in allele frequency among human ethnic groups (Fraser et al., 2012). Here we compared epigenetic differences among populations to neutral genetic variation at microsatellite loci to determine whether differences in DNA methylation among populations align with genetic drift. The correlation between microsatellite *F*_ST_ and Euclidean pairwise dissimilarity in methylation among populations (*p* = .02, *i* ^2^ = 0.19) was likely primarily driven by higher correlations between epigenetic differences at GTIIBS and Nkef and neutral genetic divergence. However, there was no significant correlation between SNP divergence and methylation pairwise dissimilarity across all eight genes (*p* = .12, *R* ^2^ = 0.064), likely due to weaker single‐gene correlations between GTIIBS and Nkef methylation and SNP divergence. While divergence in methylation among populations may be attributed in part to genetic drift, neutral genetic divergence in Chinook salmon is affected by geographic distance (Beacham et al., 2006; Heath et al., 2006). Given that geographic distance is expected to be related to ecosystem dissimilarity, it is possible that weak signals of drift may simply reflect environmental similarities among proximate populations. The weak correlation between neutral genetic markers and differences in methylation among populations suggests that while drift acts on methylation, mechanisms other than drift (such as selective mechanisms) likely also contribute to differences in methylation among populations. It is also possible that population differences in methylation are due to genetic control of methylation processes, that is, different genotypes result in different methylation patterns (Liu et al., 2012). We show that differences in methylation among populations are not well explained by genetic drift alone, suggesting that methylation is likely also influenced by a combination of genomic differences among populations, environmental acclimation, and local adaptation.

We found that ATUs (a measure of developmental timing in salmon), and the interaction between population and sampling year influenced DNA methylation. DNA methylation patterns have been shown to change through development in fish (Fang et al., 2013; Fellous et al., 2018; Venney et al., 2020), and thus, we expected differences in methylation levels in the eyed eggs as they developed. In mangrove rivulus (*Kryptolebias marmoratus*), changes in methylation occurred during development throughout organogenesis leading up to hatch (Fellous et al., 2018). However, while developmental changes in methylation are well‐characterized, interannual changes in methylation are not. We found a significant population by sampling year interaction on one gene after correcting for multiple comparisons when controlling for ATU in Harr and BQ 2015 and 2017 samples. The significant population by year effect suggests that there is some interannual variation in methylation within populations which is likely due to acclimation, though population‐level differences persist across years. These differences could be due to changes in freshwater and marine environments experienced by the parents and offspring from year to year. This raises the question of whether the egg's freshwater environment, or the parental marine and/or freshwater environments are influencing offspring methylation patterns. Since the population of origin (Harr vs. BQ) significantly affected methylation of four genes after FDR correction, and sampling year explained very little phenotypic variation in methylation (Table 3), population clearly has a greater effect on methylation state than sampling year. Our results reinforce the importance of controlling for potential confounding variables such as organism age/developmental stage and year of sampling, since methylation is a highly sensitive and dynamic mechanism for controlling gene expression.

Population epigenetic status is an important new consideration in evolutionary and ecological studies (Bossdorf et al., 2008) since DNA methylation could act as a highly dynamic evolutionary mechanism upon which selection could act (Bossdorf et al., 2008; Hu & Barrett, 2017). Unlike genetic adaptation, which requires standing variation and selection, methylation changes are rapid and dynamic, adding an additional layer of complexity and specificity for organisms to acclimate and adapt to their environment (Bossdorf et al., 2008; Hu & Barrett, 2017). In this study, we provide evidence for differences in methylation among populations of Chinook salmon, consistent with previous population epigenetic studies in other taxa (Barfield et al., 2014; Foust et al., 2016; Fraser et al., 2012; Herrera & Bazaga, 2010; Liu et al., 2012; Platt et al., 2015; Richards et al., 2010). Despite reported strong environmental effects on DNA methylation (Barfield et al., 2014; Bossdorf et al., 2008; Foust et al., 2016; Herrera & Bazaga, 2010; Richards et al., 2010), we found no link between freshwater environmental parameters and population differences in methylation. This may be due to (a) methylation corresponding to the marine environment experienced by the parents rather than freshwater variables considered here; (b) strong maternal effects on methylation at the eyed egg stage in Chinook salmon (Venney et al., 2020), which could decrease DNA methylation–environment correlations due to varying environmental experiences of individual mothers; or (c) key environmental variables that affect methylation but were not included in our analysis. We identified weak correlations between genetic drift and DNA methylation, indicating that while some changes in methylation state among populations are likely due to drift, other differences could be the result of selection (Bossdorf et al., 2008) or are linked to underlying functional genetic differences (Fraser et al., 2012).

Characterizing sources of phenotypic variation among natural populations are critical to understanding individual variation and the adaptive potential and resiliency of natural populations. DNA methylation is an important source of phenotypic variation, and a substrate for characterizing adaptive response in nature since an organism's environment and experiences can influence methylation levels (Bossdorf et al., 2008; Burggren, 2014). Furthermore, methylation signals can be passed on to offspring generations and beyond (Kamstra et al., 2018; Santangeli et al., 2019), resulting in rapid adaptation and evolutionary change in response to changing environments.

## CONFLICT OF INTEREST

The authors declare no conflict of interest.

## AUTHOR CONTRIBUTIONS

**Clare J. Venney:** Conceptualization (equal); Data curation (lead); Formal analysis (lead); Investigation (lead); Methodology (equal); Writing‐original draft (lead); Writing‐review & editing (lead). **Ben J. G. Sutherland:** Data curation (supporting); Formal analysis (supporting); Investigation (supporting); Methodology (supporting); Writing‐original draft (supporting); Writing‐review & editing (supporting). **Terry D. Beacham:** Data curation (supporting); Formal analysis (supporting); Investigation (supporting); Methodology (supporting); Writing‐original draft (supporting); Writing‐review & editing (supporting). **Daniel D. Heath:** Conceptualization (equal); Funding acquisition (lead); Investigation (equal); Methodology (equal); Project administration (lead); Resources (lead); Supervision (equal); Writing‐original draft (supporting); Writing‐review & editing (supporting).

## Data Availability

Bisulfite sequencing data, microsatellite and SNP data is available on Dryad (https://doi.org/10.5061/dryad.pnvx0k6md).
